# The Synthesis and Properties of TIPA-Dominated Porous Metal-Organic Frameworks

**DOI:** 10.3390/nano11112791

**Published:** 2021-10-21

**Authors:** Hongru Fu, Yuying Jiang, Fei Wang, Jian Zhang

**Affiliations:** 1 State Key Laboratory of Structural Chemistry, Fujian Institute of Research on the Structure of Matter, Chinese Academy of Sciences, Fuzhou 350002, China; hongrufu2015@163.com; 2 College of Chemistry and Chemical Engineering, Luoyang Normal University, Luoyang 471934, China; jiangyuyinghx@126.com

**Keywords:** Metal-Organic Frameworks, tris (4-(1H-imidazol-1-yl) phenyl) amine, porous crystalline materials

## Abstract

Metal-Organic Frameworks (MOFs) as a class of crystalline materials are constructed using metal nodes and organic spacers. Polydentate N-donor ligands play a mainstay-type role in the construction of metal−organic frameworks, especially cationic MOFs. Highly stable cationic MOFs with high porosity and open channels exhibit distinct advantages, they can act as a powerful ion exchange platform for the capture of toxic heavy-metal oxoanions through a Single-Crystal to Single-Crystal (SC-SC) pattern. Porous luminescent MOFs can act as nano-sized containers to encapsulate guest emitters and construct multi-emitter materials for chemical sensing. This feature article reviews the synthesis and application of porous Metal-Organic Frameworks based on tridentate ligand tris (4-(1H-imidazol-1-yl) phenyl) amine (TIPA) and focuses on design strategies for the synthesis of TIPA-dominated Metal-Organic Frameworks with high porosity and stability. The design strategies are integrated into four types: small organic molecule as auxiliaries, inorganic oxyanion as auxiliaries, small organic molecule as secondary linkers, and metal clusters as nodes. The applications of ratiometric sensing, the adsorption of oxyanions contaminants from water, and small molecule gas storage are summarized. We hope to provide experience and inspiration in the design and construction of highly porous MOFs base on polydentate N-donor ligands.

## 1. Introduction

Metal-Organic Frameworks (MOFs) are highly crystallized organic–inorganic hybrid materials that are formed through self-assembly of metal ions/metal clusters and organic ligands [1]. MOFs have strong structural advantages and benefit from a predesigned architecture, well-ordered porous structures, and versatile building blocks, which enable them to be sculpted into desirable materials for diverse applications [2,3,4,5,6,7,8,9].

Luminescent Metal-Organic Frameworks (LMOFs), a subclass of MOFs, are promising light-harvesting and energy transfer platforms that have been widely explored in single-phased white-light emission, optical sensors, photocatalysis, and anticounterfeiting applications [10,11,12,13,14,15,16,17,18]. MOFs have multiple photonic units, originating from inorganic metal nodes, organic chromophores, or synergistic effects of host-guest compositions. Importantly, highly porous MOFs can serve as nanocontainers to encapsulate guest emitters. Especially, benefiting from confinement and dispersion effects, the introduction of organic fluorescent dyes into MOFs can remarkably minimize nonradiative processes and Aggregation-Caused Quenching (ACQ) effects, and greatly enhanced the luminescent quantum yields [19,20,21,22]. Making full use of the encapsulation of organic luminophores and dyes, multiple emission host-guest systems can be easily prepared, which is crucial to achieve a tunable emission that contributes to ratiometric fluorescence sensing.

Cationic MOFs featuring high porosity and water stability have been demonstrated as exceptional ion-exchange platforms for the capture of toxic oxoanions and dyes. Importantly, compared with other solid porous materials, such as zeolites, activated carbons, and molecular sieves, ion exchange can be achieved via Single-Crystal to Single-Crystal (SC-SC) transformations [20,21,22,23,24,25,26,27,28,29], consequently leading to the obtaining of accurate host-guest structures to directly determine host-guest interactions and adsorption behaviors, as well as providing a new approach to reveal adsorption mechanisms.

**Figure 1:**
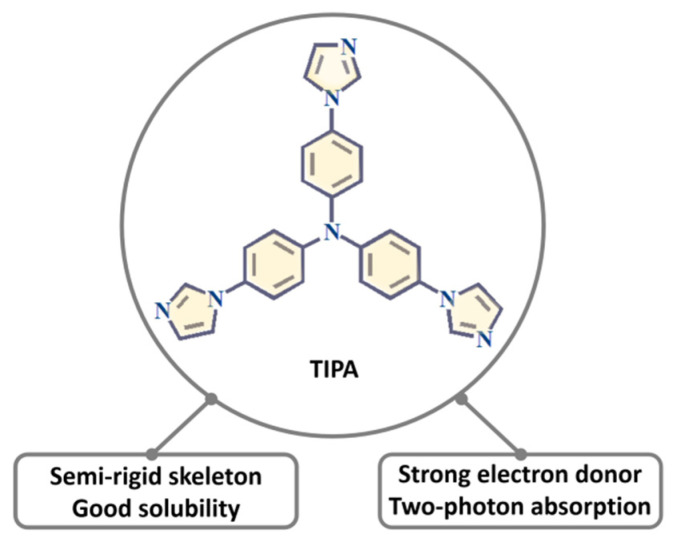
The skeleton and optical properties of TIPA.

We narrowed our interest in MOFs based on tris (4-(1H-imidazol-1-yl)phenyl) amine (TIPA), and exploited its fascinating structures and properties. Triphenylamine (TPA) has a rigid aromatic moiety and electron-donor unit, featuring two-photon absorption [30], outstanding hole mobility [31], high optical stability [32], and high molar extinction coefficient [33], and exhibits intrinsically excellent photophysical and chemical characteristics (Figure 1). Electron-withdrawing substituents, such as imidazole on the ligand, can finely modulate optical property by forming a push−pull structure with the electron-rich triphenylamine core. In addition, TIPA, as a relatively long and semi-rigid star-like structure, more easily meets the geometric requirements of metal ions. Given these facts, porous MOFs with open windows can be constructed using TIPA as the linker. Furthermore, based on the strategy of charge balance and compensation, cationic MOFs consisting of nanoscale channels can be built through the assembly of metal centers and TIPA molecules, which allows to trap toxic oxoanions. In addition, luminescent MOFs or guest-encapsulated MOFs can be prepared for fluorescence sensors. As a result, TIPA can play a significant role in crystal chemistry and coordination chemistry, further promoting the structural and functional diversity of MOFs.

In this article, we mainly focus on the synthesis and application of the highly porous MOF base with TIPA ligands. We sum up synthesis strategies for MOFs with high pores, including small organic molecule as auxiliaries, inorganic oxyanion as auxiliaries, small organic molecule as secondary linkers, and metal clusters as nodes, as well as discuss crucial factors. We also introduce some applications of highly porous frameworks, such as the capture of toxic oxoanions, luminescent detection, and the adsorption and separation of small-molecule gases. In particular, we highlight the advantage of TIPA-based MOFs as Single-Crystal containers for the encapsulation of toxic oxoanions and small molecule dyes using the SC-SC pattern. In addition, the prospect of future research in this field and the still-existing challenges are also discussed.

## 2. Construction Strategies for TIPA-Dominated Porous MOFs

The preparation of a highly porous Metal-Organic Framework is quite a complicated process and is associated with crystallization environments. More precisely, many factors, such as the organic ligands, metal ions, temperature, pH value, and the molar ratio of raw materials, can affect the formation of MOFs [34,35,36,37,38,39]. To a certain extent, organic ligands and metal nodes play a central role. TIPA, as a semi-rigid tridentate neutral ligand, has a relatively long skeleton, providing the prerequisite to construct a highly porous framework.

In the literature, using TIPA molecules as linkers for the synthesis of MOFs has been reported since 2011 [40]. The corresponding compounds were summarized in Table 1. Hundreds of TIPA-dominated MOFs have been synthesized, mainly by adopting the strategy of the mixing the ligands of TIPA molecules and various carboxylate ligands in early stages. As a whole, the great accomplishments exhibited these contributions to the construction and evolution of topological structures, especially, the fascinating polyrotaxane, polycatenation, and high-fold interpenetrating structures, have been created [41,42,43,44,45,46,47,48,49,50,51,52,53,54,55,56,57,58,59]. Nevertheless, frameworks that are highly porous and with open channels are exceedingly rare, and their properties were only focused on luminescence spectroscopy; the application and function was not developed. Until 2014, we explored a new way to promote crystal coordination chemistry of TIPA, we tried to synthesize cationic porous MOFs only using TIPA molecules as linkers, without the assistance of carboxylate ligands [60]. We took the strategy one step further, and expanded to other special routes, resulting in the realization of rigid and porous frameworks. A milestone work was reported by our group in 2015, namely, a water stable MOF FIR-53 that exhibited fast capture of Cr_2_O_7_^2−^ ions through the SC-SC approach [61]. Subsequently, dozens of TIPA-dominated MOFs with high stability and porosity were successfully prepared. Thus far, the design strategies of highly porous MOFs based on TIPA have been exploited and can be classified into four major categories: (1) small organic molecule as auxiliaries, (2) inorganic oxyanion as auxiliaries, (3) small organic molecule as secondary linkers, and (4) metal cluster as nodes.

### 2.1. Small Organic Molecule as Auxiliary

**Figure 2:**
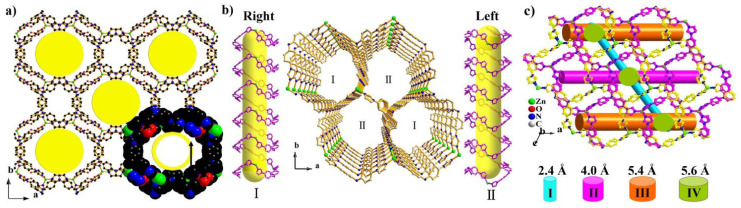
(**a**) Open framework with large channels in FIR-53. (**b**) Open framework with large channels in FIR-54. (**c**) Open framework with large channels in [Zn_2_ (TIPA)_2_ (OH) (NO_3_)_3_] 5H_2_O. Reproduced with permission [58]. Copyright 2020, the American Chemical Society.

The first TIPA-based cationic MOF with nanoscaled channels was prepared by our group [61]. When we prepared a MOF consisting of TIPA and int (isonicotinic acid), we tried to synthesize a new MOF by replacing int with L-proline, because L-proline is a chiral molecule and has a similar size to int. As a result, a new framework, namely FIR-53 (FIR = Fujian Institute Research), was synthesized. FIR-53 has a double layer structure. The *μ*_3_-TIPA ligands connect Zn^2+^ ions to yield a single layer, which is then bridged over each other to form a dual-sheet structure by Zn-OH-Zn units. The overall framework is a non-interpenetrated network with 41.6% porosity that has one-dimensional ellipse channels along the c axis, with a window size of approximately 18 × 13 Å^2^ (Figure 2a). Importantly, this framework has extremely high stability in water and air, and we can obtain an accurate structure using Single-Crystal X-ray diffraction after being exposed in water and air one year.897/*

Inspired by this, other amino acids have been used as regulators. FIR-54 was also prepared with L-proline as the regulator. FIR-54 is a 2-fold interpenetrating cationic framework, featuring one-dimensional large channels with a cross section of 10.5 × 10.5 Å^2^ (Figure 2b). Generally, these kinds of organic molecules do not appear in the final compositions of coordination compounds; perhaps they played the roles of structure direct agents (SDAs), similar to organic amines in the formation of zeolites. Another cationic framework [Zn_2_ (TIPA)_2_ (OH) (NO_3_)_3_]·5H_2_O [62] is the supramolecular isomer FIR-53. The framework contains four kinds of channels, the smallest apertures are 5.6, 5.4, 4.0, and 2.4 Å, respectively (Figure 2c). Through the assistant of glycine, [Ni (TIPA) (COO^−^)_2_ (H_2_O)]·2 (DMF)2 (H_2_O) [63] was synthesized. The framework has a one-dimensional nano-scaled channel along the c-axis with a window size of 6.3 × 7.2 Å^2^. All of these frameworks exhibited a high porosity and high stability in water, common organic solvent, and air.

### 2.2. Inorganic Oxyanion as Auxiliary

Another strategy to prepare TIPA-based porous frameworks is to adopt inorganic oxyanions as auxiliaries; the inorganic oxyanions can bridge metal center ions to yield metal-oxyanion subunits. SO_4_^2−^, PO_4_^3−^ and HPO_3_^2−^ are always selected preferentially as they are known to bind strongly with metal nodes. A water-stable cationic MOF {[Ni_2_ (L)_3_ (SO_4_)^-^ (H_2_O)_3_]·(SO_4_)·xG}n (1′-SO_4_; L = tris (4-(1H-imidazol-1-yl)phenyl) amine; G = DMF, H_2_O) with a 3D framework was reported by Ghosh [64]. The asymmetric unit contains two sulphate anions, one is connected to the Ni^2+^ ions, while the other is a free counterion in the space. The total free volume of the compound is 4592.83 Å^3^, which corresponds to 24.6% of the total volume. This framework has high hydrolytic stability. Two hybrid zincophosphites [Zn_2_ (HPO_3_)_2_ (TIPA)]·2H_2_O and [Zn_3_ (HPO_3_)_3_ (TIPA)]·6H_2_O [65] were prepared through the assembly of TIPA and phosphite, both of which feature inorganic zincophosphite chains as supramolecular building blocks.

### 2.3. Small Organic Molecule as Secondary Linker

In theory, it is reliable to use longer linkers to synthesize MOFs with larger pores; however, there are dozens of examples where MOFs were prepared using TIPA and carboxylates as colinkers, and almost no highly porous MOFs were constructed. Considering that the highly porous framework, FIR-53, was assembled through the stacking of [Zn_2_ (TIPA) (OH)]^3−^ layers, small molecule imidazole and imidazole and its derivatives can also adopt a μ_2_-coordination mode which is similar to -OH. On the basis of this strategy, two MOFs, namely, [Zn_3_ (TIPA)_2_ (Im)_3_]·3NO_3_·solvent (Im = imidazole) with a three-dimensional (3D) pillared-layer framework and [Zn_2_ (TIPA)_2_ (Tz)][Zn(TIPA) (NO_3_) (H_2_O)]·4NO_3_·3DOA·solvent (Tz = tetrazole, DOA = 1,4-dioxane) [66] with a rare sandwich structure were synthesized. The former compound has a kind of large elliptic channel, with pore apertures of 7.7 and 13.1 Å along the a axis, and the free NO_3_^−^ ions are located inside the channels. In the structure view of the latter compound, each Zn^2+^ ion adopts the four-connected model, linked by three TIPA molecules to generate a single layer, further bridged by μ_2_-Zn-Tz-Zn units to form a bilayer structure with an open window of ca. 11.0 Å (Figure 2c). These two compounds have a porosity of 32.6% and 28.8%, respectively.

### 2.4. Metal Clusters as Nodes

Using polynuclear clusters, especially, high-nuclear clusters, such as hexa-, octa-, dode-nuclear and greater, as building units has been demonstrated as a feasible strategy to construct highly porous MOFs [73,74,75,76]. To some extent, polynuclear clusters can endow highly-connected numbers and further decrease steric hindrance of organic ligands around them. Rationally selecting the origin of clusters and modulators of synthesis processes could promote the synthesis of targeted structures.

**Figure 3:**
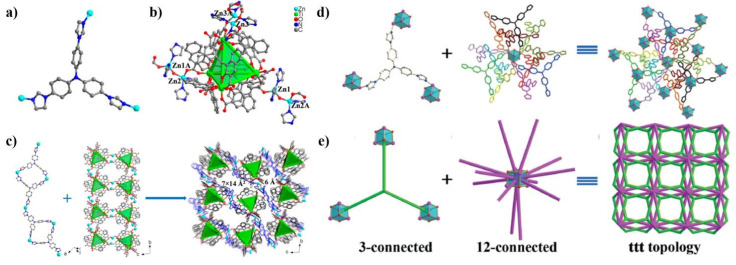
(**a**) Coordination environment of TIPA ligand. (**b**) Coordination model of the ZnII atoms, and Ti_4_L_6_ cage in PTC-220. (**c**) Zn atoms linking the TIPA ligands into a chain. and Zn atoms linking the Ti_4_L_6_ cages into a ladder-shaped chain, 3D framework of PTC-220 with different pore sizes. Reproduced with permission [75]. Copyright 2020, the American Chemical Society. (**d**) A 3-connected TIPA ligand and simplified 3-connected linker. (**e**) A 12-connected [Cd_4_ (μ_3_-OH)_4_] cubic cluster and simplified 12-connected node, (3,12)-connected framework and the ttt topology. H atoms removed for clarity. Reproduced with permission [67]. Copyright 2014, Royal Society of Chemistry.

Sun’s group reported an unprecedented (3,12)-connected MOF, [Cd (TIPA) (μ_3_-OH)·NO_3_·EtOH·DMF]n [67] with a ttt topology based on cubic [Cd_4_(*μ*_3_-OH)_4_] clusters and TIPA ligands (Figure 3d,e). Each Cd^2+^ ion adopted an octahedral configuration coordinated by three nitrogen atoms from three independent TIPA molecules and three oxygen atoms from three independent μ_3_-OH^−^. Four crystallographically equivalent Cd (II) ions are bridged by four μ_3_-OH^−^ ions to yield cubic and 12-connected [Cd_4_ (μ_3_-OH)_4_] clusters where Cd and O alternatively located at the corners. The *μ*_3_-coordinated TIPA ligands connected with [Cd_4_ (μ_3_-OH)_4_] clusters to form a three-dimensional framework with moderate pores. A stepwise assembly strategy was established for the construction of Ti-MOFs by our group. Particularly, the combination of Ti_4_L_6_ (L = embonate) cages, metal ions and our ligands provide an efficient approach to prepare Ti_4_L_6_-cage-based MOFs. By selecting TIPA as auxiliary ligands, a network of PTC-220 (Me_2_NH_2_)_3_ [(Ti_4_L_6_)Zn_3_ (OH) (TIPA)_2_]· guests [68] was constructed (Figure 3a–c). In PTC-220, the ligand adopts a μ_3_-coordinated mode to connect Zn^2+^ ions into an one-dimensional chain, Ti_4_L_6_ cages are bridged Zn^2+^ ions that form a ladder-shaped chain. These two kinds of chains are mutually crosswise arranged to result in a three-dimensional framework, and this framework consists of two types of channels with relative dimensions of 7 × 14 Å^2^ and 6 × 6 Å^2^, respectively (Figure 3d). Our group also prepared a porous framework [Cd (TIPA)Cl_2_]·2 (DMF)·H_2_O [60] based on a 6-connected Cd_2_Cl_4_ cluster. The assembly of Cd (II) ions and TIPA molecules generated a three-dimensional framework with large channels. The isolated frameworks are further joined by *μ*_2_-Cl^−^ ions to form a double-walled network. The final 2-fold interpenetrating structure has a solvent-accessible void space of 33.0% of the total crystal volume.

## 3. The Application of TIPA-Dominated Porous MOFs

### 3.1. The Capture of Toxic Oxoanions

**Figure 4:**
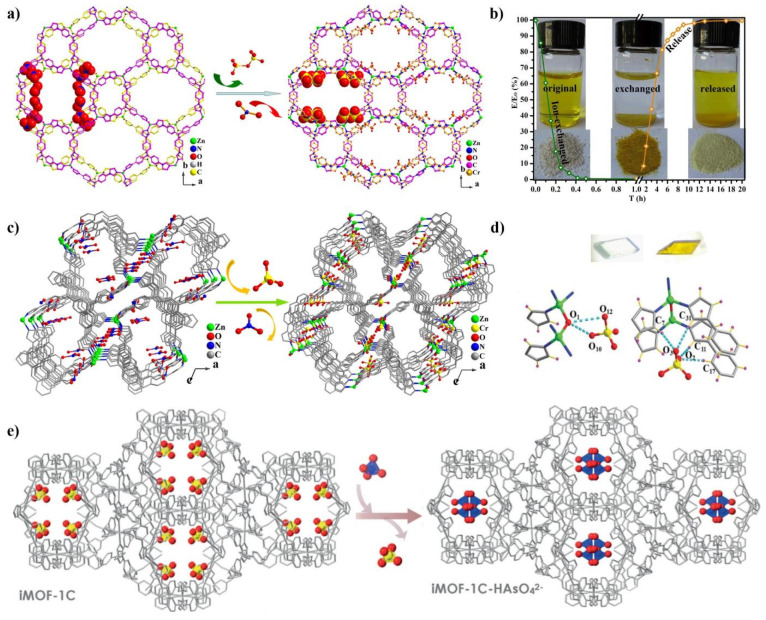
(**a**) Structures of FIR-53 before and after ion exchange: 3D structure of FIR-53 containing NO_3_^−^ along the c axis, 3D structure of FIR-53 containing Cr_2_O_7_^2−^ along the c axis. (**b**) Photographs show the color of crystals before and after trapping−releasing process. Reproduced with permission [58]. Copyright 2015, the American Chemical Society. (**c**) A 3D structure of compound [Zn_2_ (TIPA)_2_ (OH) (NO_3_)_3_]·5H_2_O along the b-axis: NO_3_^−^ ions as guests in the channels, CrO_4_^2−^ ions instead of NO_3_^−^ located in the channels. (**d**) H bonds between the framework and CrO_4_^2−^, color change of FIR-53 crystals before and after ion exchange. Reproduced with permission [62]. Copyright 2018, Royal Society of Chemistry. (**e**) Packing diagram representing Single-Crystal-to-Single-Crystal transformation of parent cationic MOF, iMOF-1C, to HAsO_4_^2−^ encapsulated phase. Reproduced with permission [67]. Copyright 2020. John Wiley & Sons, Ltd.

Several cationic frameworks with high porosity and high stability were prepared using TIPA as linkers. Reported by our group, two cationic MOFs (FIR-53 and FIR- 54) [58] with nanoscale channels exhibited that high capacity of chromium exceeding 100 mg g^−1^ compounds also displays fast and efficient trapping-releasing processes. Importantly, the single crystal of the chromium-loaded sample was also subject to Single-Crystal X-ray diffraction. It was found that Cr_2_O_7_^2−^ ions existed in the double layer instead of NO_3_^−^ anions. In addition, another cationic framework [Zn_2_ (TIPA)_2_ (OH) (NO_3_)_3_]·5H_2_O [62] with the same formula as FIR-53, also acted as a single crystal container to trap Cr (VI)-oxyanions in a SC-SC fashion (Figure 4c,d). It exhibited a fast sorption kinetics toward Cr (VI)-oxyanions in water with high selectivity, as well as good repeatability. Interestingly, CrO_4_^2−^ rather than Cr_2_O_7_^2−^ was confirmed in the channel from the single crystal structure. This was mainly ascribed to the spatial confinement of the channels. It was revealed that the electrostatic interaction and rich hydrogen bonds between the CrO_4_^2−^ ions and the framework played an important role in the capture of CrO_4_^2−^ ions. In addition, FIR-54 also has the high-performance adsorption behaviors of Cr (VI)-oxyanions in aqueous solutions, although the crystals remains intact after ion exchange, the existence of counterions could not be found from the Cr (VI)-oxyanion-exchanged samples, and only the accurate structure of host framework was obtained by Single-Crystal diffraction. Compared the sorption results of these three compounds, it could be determined that the size effect of the pores played a crucial role in the existence of the formation of Cr (VI)-oxyanions, largely affecting the aggregate state. These results are remarkably important to prepare practical materials for Cr (VI)-oxyanion capture.

Ghosh et al. reported a water-stable cationic MOF {[Ni_2_ (L)_3_ (SO_4_)-(H_2_O)_3_]·(SO_4_)·xG}n (1′-SO_4_; L = tris (4-(1H-imidazol-1-yl)phenyl) amine; also referred to as iMOF-1C, G = guest solvent molecules and C = Cationic) (Figure 4e) [69]. MOF 1-SO_4_ can act as dual adsorbent for permanganate ions and dichromate. This compound exhibits a moderate capacity of about 166 mg g^−1^ for Cr_2_O_7_^2−^ ions. Although both the crystallinity and integrity of the framework after the exchange was retained, the crystal structure of 1-SO_4_ after saturated adsorption by SC-XRD was unsuccessful due to the poor diffraction. Meanwhile, this group used iMOF-1C as an efficient ion-exchanger to capture the toxic oxoanions selenium (SeO_4_^2−^) and arsenic (HAsO_4_^2−^) from an aqueous medium. Interestingly, it exhibited fast kinetics and high sorption capacity, 100 mg g^−1^ and 84 mg g^−1^ for SeO_4_^2−^ and HAsO_4_^2−^, respectively, which are among the highest values reported in the field of MOFs. A Single-Crystal structure, after arsenate and selenate oxoanion, could be obtained, monohydrogen arsenate and selenate anion replace the sulfate anion along the 1D channel of the framework. This result could be attributed to the effect of the charge and geometry, where the SeO_4_^2−^ and HAsO_4_^2−^ anions are tetrahedral and have similar shapes and sizes to SO_4_^2−^. The adsorption of iMOF-1C provided a typical example to reveal the exchange mechanism for the removal of hazardous toxic oxoanions. Our group reported a two-dimensional cationic framework, namely [Cd (TIPA)_2_] (ClO_4_^−^)_2_]·(DMF)3 (H_2_O) [62]; it was employed an ion-exchange material for the capture of Cr_2_O_7_^2−^ anions. The capture capacity of this compound was up to 116.6 mg g^−1^. This compound also showed excellent selectivity, and the adsorption ability of Cr (VI) was not significantly affected, in addition, the concentration of various types of anion species was five times higher than that of the Cr_2_O_7_^2−^ anion. Importantly, it has the excellent reusability. The guest-loaded MOFs were immersed in KNO_3_ aqueous solution, up to 92% Cr_2_O_7_^2−^ anions were released again into the solutions after 24 h. This compound maintained approximately 81% release efficiency during five continuous cycles. In the work of Wang′s group, an isomorphic structure was reported using Ni^2+^ ions as nodes [70]. It exhibited high alkaline stability and exceptional ^99^TcO4−capture selectivity. Liquid scintillation counting (LSC) measurements verified that this compound exhibited extremely rapid kinetics with an equilibrium time of ~5 min; this can be largely attributed to the nature of the layered structure, which is in favor of the diffusion and transportation of anions. Moreover, the high positive charge density and hydrophobicity also increased the affinity of the 2D layers for ^99^TcO_4_^−^ anions. In addition, similar exchange kinetics were found for ReO_4_^−^ anions. The maximum anion-exchange capacity of the ReO_4_^−^ anions was 318 ± 8 mg/g. Of significance, this compound exhibited fairly high adsorption amounts of ReO_4_− removal, even SO_4_^2−^ was present in a 6000-fold excess. These results make it an extremely viable candidate for the selective removal of ReO_4_^−^/^99^TcO_4_^−^ from waste solutions.

### 3.2. Sensing

MOFs as a class of luminescent materials largely depend on ligands and metal nodes. The powder of TIPA itself has weak blue emission; however, the emission intensity is greatly enhanced via crystallization, where the orientation and arrangement of TIPA molecules was improved and the rigidity was increased. In Fu′s contribution, [Cd (TIPA)_2_] (ClO_4_^−^)_2_]·(DMF)3 (H_2_O) was used as a luminescent probe to detect anions in aqueous media 62. This compound exhibited high selectively against Cr_2_O_7_^2−^ anions among (BF_4_^−^, VO_4_^3−^, MoO_4_^2−^, WO_4_^2−^, ClO_4_^−^, SO_4_^2−^, NO_3_^−^, Br^−^, Cl^−^, I^−^, SCN^−^, OAc^−^ and OX_2_−). The K_sv_ value was calculated to be 7.15 × 10^4^ L mol^−1^ and the detection limit was as low as 8 ppb (S/N = 3), which matched that of the reported MOFs. Guo et al. prepared self-interpenetrating MOFs [CdL (NO_3_^−^)_2_·4H_2_O]_n_ (L = tris (4-(1H-imidazol-1-yl)phenyl) amine) [77], which has a high water stability. This compound was implemented as luminescent sensors to monitor antibiotics in aqueous solutions. This MOF has excellent selectivity towards NFT and a Ksv value of NFT as high as 4.64 × 10^4^ M^–1^.

**Figure 5:**
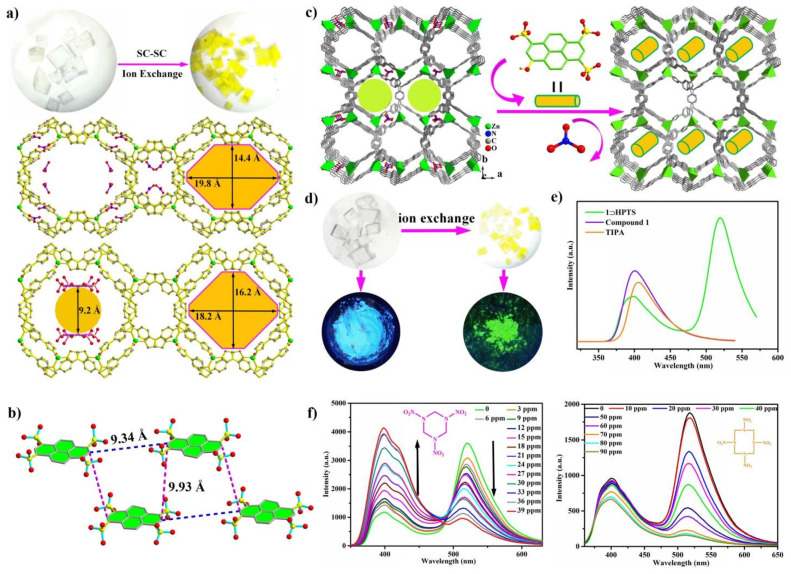
(**a**) Photograph of FIR-53 crystals, 3D framework of FIR-53 along the c axis, the window size from 19.8 × 14.4 Å^2^ of FIR-53 to 20.2 × 17.3 Å^2^ of SG7@FIR-53 along c axis. (**b**) Photograph of FIR-53 after ion exchange, the arrangement and configuration of SG73− along the two sides of the channels. Reproduced with permission [69]. Copyright 2018, the American Chemical Society. (**c**) A 3D framework of compound FIR-54 after ion exchange. (**d**) Photographs show the colors of crystals before and after the trapping process in the two images above, and the fluorescence emission of the crystal powder before and after ion exchange under ultra-violet light in the two images below. (**e**) Photographs show the solid-state fluorescence emission of compound FIR-54, TIPA, and MOFÉHPTS. (**f**) The fluorescence titrations of MOFÉHPTS upon exposure to the aqueous solution of RDX and HMX. Reproduced with permission [79]. Copyright 2018, Royal Society of Chemistry.

Beyond this, the inherent porous nature of MOFs enables the encapsulation of versatile chromophore guests into the MOF pores to construct multiple-luminescence MOFs. For example, our group selected SG7 (solvent green 7, a sulfonated pyrene) as a guest with green emissions as the precursors of FIR-53 to prepare SG7@FIR-53 via an ion-exchange route [71]. SG7 is a normal dye molecule for fluorescence chemistry. The parent MOF exhibited ultrahigh stability and the crystallinity and morphology maintained excellent integrity in water or air for at least six months during the ion-exchange period. The color of the crystals gradually became transparent to yellow green. SG7@FIR-53 showed dual emission at 375−455 and 500−550 nm under the single excitation wavelength at 310 nm, while the free TIPA ligand and FIR-53 displayed similar blue emissions in the range from 375 to 470 nm, indicating that SG7 was introduced inside the channels and existed in the form of excimer aggregation; by comparison, the monomer emission of SG7 (pyrene) was during 375–405 nm [78]. In order to verify this hypothesis of excimer aggregation, we attempted to obtain the crystal structure of the guest-loaded framework. We determined up the dye-exchanged MOF when the ion-exchange process lasted a month. Fortunately, the structure of the SG7-encapsulated FIR-53 was successfully obtained via the Single-Crystal X-ray diffraction method. SG7^3−^ instead of NO_3_^−^ anions located on the sides of the channels further formed a paralleled double layer in the direction of the c axis; the distance of the adjacent SG7^3−^ ions was approximately 10 Å (Figure 5a,b). The stacking of the SG7^3−^ anions indicated that it yielded the excimer emission of pyrene, which is coincident with the fluorescence spectra analysis. To the best of our knowledge, it is the first report where the introduction of dye into MOF was achieved via SC-SC transformation. Different anions were detected in aqueous solution though the fluorescence changes of SG7@FIR-53; it was found that Cr_2_O_7_^2−^ or MnO_4_^−^ showed a marked quenching effect on SG7@FIR-53 and the detection limit was calculated as low as 1.02 and 0.12 ppb, respectively.

We also introduced 8-hydroxy-1,3,6-pyrenetrisulfonicacid trisodium salt (HPTS) into a cationic MOF [Zn (TIPA) (NO_3_^−^) (H_2_O)]·5H_2_O, which has the same framework as FIR-54. HPTS powder has no fluorescence owing to the Aggregation-Caused Quenching effect; however, MOF⊃HPTS show the bright green emission [79]. By fluorescence measurement, the complex MOF⊃HPTS shows a dual-emitting behavior at around 375–450 and 500–575 nm, belonging to the blue emission of TIPA and the green emission of HPTS, respectively (Figure 5c–e). It is worth mentioning that MOF⊃HPTS showed an intensive emission with only a slight reduction after 60 days in air and aqueous solution, respectively. This result indicated that the confinement effect of the porous network could protect the aggregation state of fluorescent dyes and effectively suppress Aggregation-Caused Quenching. MOF⊃HPTS can serve as a durable dual-emitting sensor. MOF⊃HPTS exhibited the obvious quenching effects towards a wide range of aromatic nitro-explosives in aqueous solution. If fluorescence emission of HPTS is the reference, the concentrations of TNP, 2-NP, 3-NP, and 4-NP caused the complete quenching of MOF⊃HPTS as low as 50 ppm. Importantly, MOF⊃HPTS showed high sensitivity to aliphatic nitro-compounds. DMNB (2,3-dimethyl-2,3-dinitrobutane) and RDX (1,3,5-triazine) and HMX (cyclotetramethylenetetranitramine) caused almost total quenching of HPTS at a low concentration below 100 ppm. Especially, MOF⊃HPTS exhibited a ratiometric fluorescence sensing for RDX detection, with increasing concentration of RDX, the emission intensity of HPTS decreased sharply, while the blue emission at 400 nm was greatly strengthened (Figure 5f). So far, it is the only example among luminescent sensors that has the ratiometric fluorescence effect against RDX. In addition, MOF⊃HPTS exhibits a high response to a broad class of nitro-containing antibiotics.

### 3.3. Gas Adsorption

**Figure 6:**
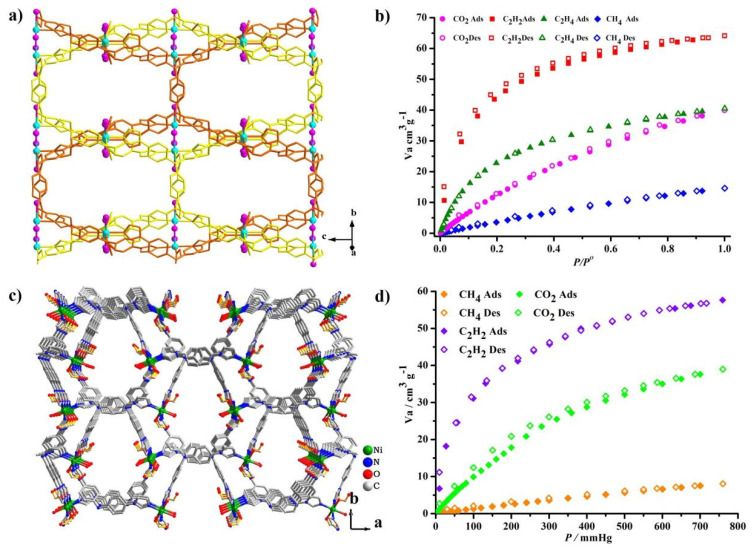
(**a**) A simple framework containing two Cd-TIPA frameworks linked by *μ*_2_-Cl− ions. (**b**) C_2_H_2_, C_2_H_4_, CH_4_ and CO_2_ sorption isotherms at 297 K. Reproduced with permission [60]. Copyright 2014, the American Chemical Society. (**c**) 3D framework of compound [Ni (TIPA)(COO^−^)2(H_2_O)]·2(DMF)2(H_2_O) along the c axis. (**d**) adsorption isotherms of CO_2_, CH_4_ and C_2_H_2_ at 297 K. Reproduced with permission [63]. Copyright 2018, Royal Society of Chemistry.

The high stability and the rich porous environment of these TIPA-based frameworks enabled these MOFs to serve as excellent adsorbents for the storage and purification of gas molecules. Our group reported a nonporous MOF with gas adsorption selectivity, [Cd (TIPA)Cl_2_]·2 (DMF)·H_2_O [60], which has a two-fold interpenetrated framework composed of a dinuclear Cd_2_Cl_2_ unit and TIPA. This compound displays the typical type I sorption of N_2_ with a BET surface area of 348.8 m^2^ g^−1^. This compound has a moderate adsorption capacity of C_2_H_2_ (64.13 cm^3^ g^−1^) and C_2_H_4_ (40.52 cm^3^ g^−1^) and CH_4_ (12.04 cm^3^ g^−1^) at 297 K and 1 atm (Figure 6a,b); however, the Qst (adsorption isosteric heat) values of C_2_H_2_ and C_2_H_4_ were 41.05 and 34.69 kJ/mol, respectively, indicating strong solid-gas interactions between the framework and the small gas molecules. As a result, the adsorption selectivity of C_2_H_2_/CH_4_ and C_2_H_4_/CH_4_ was 39.1 and 13.5 at 297 K, respectively. A 2D + 2D → 3D interpenetrating framework [Ni(TIPA)(COO−)_2_(H_2_O)]·2(DMF)2(H_2_O) [63] was prepared for small hydrocarbon separations. The surface area was 404.6 m^2^ g^−1^ measured by the CO_2_ adsorption at 195 K. This porous material exhibited moderate capacity of C_2_H_2_ and CO_2_, 65.8 and 46.9 cm^3^ g^−1^ at 273 K, 56.8 and 39.0 cm^3^ g^−1^ at room temperature (Figure 6c,d). The adsorption selectivity of C_2_H_2_/CH_4_ and C_2_H_2_/CO_2_ (equimolar binary mixtures) was calculated using the ideal adsorption solution theory (IAST) at 273 and 297 K, the selectivity of C_2_H_2_/CH_4_ and C_2_H_2_/CO_2_ in this compound is 112.2 and 8.0 at 297 K, and 228.6 and 6.1 at 273 K. The C_2_H_2_/CH_4_ selectivity is the highest one among MOFs-based materials to date [80] and was largely attributed to the confined ultra-microporous channel (the channel size was 6.3 × 7.2 Å^2^) and the naked carboxylic group, which enhanced the interactions with C_2_H_2_ molecules.

## 4. Conclusions

In this article, different methods to prepare highly porous MOFs with high stability using neutral nitrogen-based ligand TIPA, without carboxyl ligands, was briefly introduced. These porous crystalline materials have been successfully applied in the adsorption of toxic oxoanions, luminescent sensing, including cations, anions, aromatic explosives, and aliphatic nitro-compounds, and the storage and purification of small molecule gases.

TIPA as linkers, exhibits huge advantages in constructing porous MOFs; they can, not only be used to build highly stable and porous frameworks to capture several kinds of toxic oxoanions (CrO_4_^2−^, Cr_2_O_7_^2−^, SeO_4_^2−^ and HAsO_4_^2−^) in aqueous media via the SC-SC approach, but they can also be used to synthesize porous host frameworks to encapsulate guest emitters to create multiple emitting systems [81,82,83].

Although TIPA-based MOFs materials have made great achievements in synthesis and in various applications, some challenges remain to be critically solved. For example, more novel strategies were exploited to enlarge the scope of MOFs with high stability and porosity. Perhaps it is an efficient method to construct highly porous MOFs through a combination of various Ag clusters. In addition, the triphenylamine core of the ligand is a typical octupolar nonlinear optical chromophore and is known for two photon absorption-upconverted emission [84]. It is possible that TIPA-based MOFs can supply a new platform for the development and application of polarized light materials and upconversion materials. Through the preparation of chiral MOFs, new materials for circularly polarized luminescence can be provided. In recent years, phosphorescence has shown unique properties, such as a long lifetime, large stoke shift, and abundant excited states, and has been a hotspot in the optoelectronic filed [85]. TPA derivatives have been widely used to develop purely organic phosphorescence [86,87,88,89], and one TIPA-based MOF showing the phosphorescence property was reported in 2014 [72]; it is still the only one reported to this day. If phosphorescence is efficiently integrated into MOFs, more interesting and significant applications can be promoted. Despite the fact that several MOFs were selected as host platforms for the encapsulation or capture of some guests, this work focused primarily on toxic oxoanions and organic small molecules, those of other structures, such as carbon dots and perovskite dots, have not been explored. More efforts are consequently essential to prepare these kinds of host-guest materials, with the introduction of carbon dots and perovskite dots into rigid matrices, and will provide a lot of other new functions (e.g., room temperature phosphorescence, light-emitting diodes, and laser). We believe that, with further in-depth research and innovation, a bright future for TIPA-based porous MOFs can be expected.

## Figures and Tables

**Table 1:** Examples of MOFs based on TIPA.

MOF	Dimension	Porosity	Auxiliary Ligand	Ref.
[Cd (TIPA)·(NO_3_)_2_·5H_2_O] _n_	2D	28.7%		[40]
[Co (TIPA)_1/3_·Cl_3_·2H_2_O] _n_	2D	52.8%		
[Co (TIPA)·(5-OH-bdc)_3_·2H_2_O] _n_	2D	none		
[Cd_2_(TIPA)_2_·(5-OHbdc)_2_·5.5H_2_O] _n_	3D	34.4%		
[Ag_0.52_Na_0.48_(*β*-Mo_8_O_26_) (H_2_O)] [Ag_3_(Tipa)_2_]	3D	none		[41]
[Ag_6_(Tipa)_4_(*β*-Mo_8_O_26_)] [H_2_(β-Mo_8_O_26_)] 5H_2_O	2D	none		[42]
[Ag_3_(OH)(H_2_O)_2_(Tipa)_2.5_] [Mo_2_O_7_]_3_·4.5H_2_O	3D	none		
[Cd (Tipa)(L1)_2_] H_2_O	2D	none	HL1 = benzoic acid	[43]
[Cd (Tipa)(L2)] H_2_O	2D	none	H_2_L2 = 5-NH_2_-1,3-benzenedicarboxylic acid	
[Cd (Tipa)(L2)] CH_3_OH·H_2_O	3D	none		
Cd (Tipa)(L3) (H_2_O)	3D	none	H_2_L3 = 2-(4-carboxybenzylamino) benzoic acid	
[Mn (Tipa)(L2)] H_2_O	2D	none		
[Ni_2_(Tipa)_2_(L4) (H_2_O)_2_]2·Cl_4_·4H_2_O	2D	none	H_2_L4 = 1,4-benzenedicarboxylic acid	
[Ni_2_(Tipa)_2_(L5) (H_2_O)_4_] (H_4_L5) 0.5H_2_O	2D	none	H_4_L5 = 1,2,4,5-benzenetetracarboxylic acid	
{[Zn (TIPA)(mal)_1/2_] (NO_3_)·3H_2_O}_n_	3D	41.9%	mal = malonic acid	[44]
{[Zn (TIPA)(glu)_1/2_] (NO_3_)·H_2_O}_n_	3D	27.2%	glu = glutaric acid	
{[Co (TIPA)(*trans*-chdc) (H_2_O)]·H_2_O}_n_	2D	none	*trans*-chdc = *trans*-1,4-cyclohexanedicarboxylic acid	[45]
{[Ni (TIPA)(*trans*-chdc) (H_2_O)]·H_2_O}_n_	2D	none		
{[Co (TIPA)(seb)_1/2_] (NO_3_)·H_2_O}_n_	2D	none	H_2_seb = sebacic acid	
{[Ni (TIPA)(seb)_1/27_](NO_3_)·H_2_O}_n_	2D	none		
{[Zn_2_ (TIPA)(btc)(μ2-OH)]·4H_2_O}_n_	2D	none	H_3_btc = 1,3,5-benzenetricarboxylic acid	
{[Cd (TPPA)(*trans*-chdc)]} _n_	3D	none	*trans*-H_2_chdc = *trans*-1,4-cyclohexanedicarboxylic acid	[47]
{[Co (TPPA)_2_(D-ca)_2_]·(H_2_O)}_n_	2D	none	d-H_2_ca = d-camphor acid	
{[Ni (TPPA)(bdc)(H_2_O)]·(H_2_O)_4_}_n_	3D	none	H_2_bdc = benzene-*p*-dicarboxylic acid	
{[Ni (TPPA)(*trans*-chdc)(H_2_O)]·(H_2_O)_4_}_n_	3D	none		
[Co_2_(TPPA)_2_(1,3-bdc)_2_(H_2_O)] *_n_*	2D	none	1,3-H_2_bdc = 1,3-benzenedicarboxylic acid	[50]
[Zn (TPPA)(1,3-bdc)] *_n_*	2D	none		
[Zn_6_(TPPA)_2_(betc)(Hbetc)_2_(H_2_betc) (H_2_O)_6_·7H_2_O·2DMA]*_n_*	3D	24.2%	betc = 1,2,4,5-benzenetetracarboxylic dianhydride	
[Cu (TPPA)(NO_3_)_2_(H_2_O)]·2H_2_O]*_n_*	2D	none		
{[Cd (DIMPPA)(5-OH-bdc)](H_2_O)}*_n_*	2D	none	5-OH-H_2_bdc = 5-hydroxyisophthalic acid	[51]
{[Co (DIMPPA)(5-OH-bdc)](H_2_O)}*_n_*	2D	none		
{[Cd_2_(MIDPPA)_2_(D-ca)_2_(H_2_O)_2_] (H_2_O)_5_} *_n_*	3D	none	D-H_2_ca = D-camphoric acid	
{[Co_1.5_(TTPA)(BTC) (H_2_O)]_2_·13H_2_O} *_n_*	3D	51%	H_3_BTC = 1,3,5-benzenetricarboxylic acid	[52]
[Co (TTPA)(PA)] *_n_*	3D	none	H_2_PA = phthalic acid	
{[Co (TTPA)(BDA)_0.5_(NO_3_)]·3H_2_O}*_n_*	3D	27.2%	H_2_BDA = (1,1′-biphenyl)-4,4′-dicarboxylic acid	
{[Co_2_(TTPA)_3_(OBA)_2_(H_2_O)_3_]·2CH_3_CN·4H_2_O} *_n_*	2D	none	H_2_OBA = 4,4′-oxydibenzoic acid	
{[Co (TTPA)(AIP) (H_2_O)]·2H_2_O} *_n_*	2D	none	H_2_AIP = 5-aminoisophthalic acid	
{[Co (TTPA)(MIP) (H_2_O)]·2H_2_O} *_n_*	2D	none	H_2_MIP = 5-methylisophthalic acid	
{[Cd(tipa)_2_]·(ClO_4_)_2_} _n_	2D	41.9%		[54]
{[Cd(tipa)(NO_3_)_2_]} _n_	1D	33.2%		
{[Cd_2_(SO_4_)_2_(tipa)_2_]} _n_	2D	28.3%		
{[Cd(tipa)(NO_3_) (SA)]·(DMF)} _n_	2D	none	sulfanilic acid	
{[Cd(tipa)(HCOO)_2_]·*x*G} _n_	3D	25.3%		
{[Zn (TIPA)pim_0.5_]2H_2_O·NO_3_} *_n_*	3D	26.6%	H_2_pim = pimelic acid	[55]
{[Zn (TIPA)(pim)]_3_H_2_O} *_n_*	2D	38.4%		
{[WOS_3_Cu_3_Br (TIPA)]-(H_2_O)(DMF)} *_n_*	3D	29.8%		[56]
[Zn_4_ (Tipa)_4_Cl_4_]·4(G1)	3D	none		[57]
[Zn_4_(Tipa)_4_Cl_4_]·4(G2)	3D	none		
[Zn_2_(Tipa)_2_Cl_2_]·(G3)	3D	none		
[Zn_2_(Tipa)_2_(OH)]·(G4)	3D	none		
[Zn_3_(Tipa)_2_(OH)_3_]·(G5)	3D	none		
[Zn_3_(Tipa)_2_F_2_(H_2_O)_4_]·2(G6)	3D	none		
[Cd (TIPA)(suc)_0.5_(NO_3_)·½H_2_O]_n_	3D	none	succinic acid	[59]
[Ni (TIPA)(tda)_0.5_(H_2_O)·¼H_2_O]_n_	3D	none	2,5-thiophenedicarboxylic acid	
[Cd (TIPA)(tda)_0.5_·½H_2_O]	3D	none		
{[Zn (TIPA)(seb)_0.5_](NO_3_)·3.5H_2_O}_n_	3D	none	seb = sebacic acid	
[Zn_2_(Tipa)(4,4′-bpdc)_1.5_(H_2_O)(NO_3_)]·2(DMF)·H_2_O	3D	none		[60]
[Cd (Tipa)Cl_2_]·2(DMF)·H_2_O	3D	33.0%		
[Co (Tipa)Cl_2_(H_2_O)]·DMF·H_2_O	2D	none		
[Zn_2_(Tipa)_2_(OH)]·3NO_3_·12H_2_O	2D	41.6%		[61]
[Zn_2_(Tipa)_2_(OH)]·NO_3_·Cr_2_O_7_·5H_2_O	3D	none		
[Zn(Tipa)]·2NO_3_·DMF·4H_2_O)	3D	41.8%		
[Zn_2_(TIPA)_2_(OH)(NO_3_)_3_]·5H_2_O	3D	49.0%		[62]
[Zn_2_(TIPA)_2_(OH)(Cr_2_O_7_)_1.5_]·3H_2_O	3D	none		
[Ni (TIPA)(COO^−^)_2_(H_2_O)]·2(DMF)_2_	2D	39.1%		[63]
[Cd(TIPA)_2_(ClO_4_^−^)_2_]·(DMF)_3_(H_2_O)	2D	50.5%		
[{Ni_2_(TIPA)_3_(SO_4_)(H_2_O)_3_}·(SO_4_)·x(G)]_n_	3D	24.6%		[64]
[Zn_2_(HPO_3_)_2_(TIPA)]·2H_2_O	2D	none		[65]
[Zn_3_(HPO_3_)_3_(TIPA)]·6H_2_O	1D	none		
[Zn_3_(Tipa)_2_(Im)_3_]·3NO_3_·solvent	3D	28.8%	Im = imidazole	[66]
[Zn_2_(Tipa)_2_(Tz)][Zn(Tipa)(NO_3_)(H_2_O)]·4NO_3_·3DOA	2D	32.6%	Tz = tetrazole, DOA = 1,4-dioxane	
[Cd(tipa)(*μ*_3_-OH)·NO_3_·EtOH·DMF]_n_	3D	none		[67]
(Me_2_NH_2_)_3_[(Ti_4_L_6_)Zn_3_(OH)(tipa)_2_]·Guests	3D	36.2%	L = embonate	[68]
{[Ni_2_(L)_3_(SO_4_)-(H_2_O)_3_]·(HAsO_4_)·xG}n	3D	none		[69]
[Ni(tipa)_2_](NO_3_)_2_	2D	none		[70]
[Zn_2_(tipa)_2_(OH^−^)](NO_3_^−^)(SG7)_2/3_·5H_2_O	3D	none	SG7 = solvent green 7	[71]
{[Zn(tipa)Cl]·NO_3_·2DMF}_n_	3D	37.4 %		[72]
{[Cd_2_(tipa)_2_Cl_4_]·6DMF}_n_	3D	none		
{[Zn_2_(tipa)_2_Cl_2_]·2I_3_·2 DMF}_n_	3D	none		
{[Cd_2_(tipa)_2_Cl_2_(dmf)_2_]·2I_3_·4 DMF}_n_	3D	none		

## Data Availability

The data presented in this study are available in the cited references.
